# Mindful and Intuitive Eating: A Bibliometric Analysis of Research Trends From 2004 to 2024

**DOI:** 10.1111/obr.70165

**Published:** 2026-05-21

**Authors:** Mengying Zhang, Angeliki Bogossian, Emma Stanmore, Trudi Edginton, Siobhán O′Connor

**Affiliations:** ^1^ School of Health Sciences The University of Manchester Manchester UK; ^2^ School of Health and Psychological Sciences City St George's, University of London London UK; ^3^ Florence Nightingale Faculty of Nursing, Midwifery and Palliative Care King's College London London UK

**Keywords:** bibliometrics, data visualization, intuitive eating, mindful eating, mindfulness, obesity, self‐compassion

## Abstract

**Introduction:**

Excessive body weight and problematic eating behaviors are major public health concerns. Mindful eating and intuitive eating offer alternative approaches to dieting by encouraging individuals to focus on internal hunger and satiety cues. This bibliometric review aimed to analyze research trends related to mindful and intuitive eating.

**Methods:**

We searched Scopus to identify relevant research from 2004 to 2024, with titles and abstracts screened for relevancy. We used VOSviewer and Bibliometrix to extract data and produce bibliometric analyses.

**Results:**

Research (*n* = 1064) on mindful and intuitive eating increased most years, with 2023 (*n* = 143, 13.44%) and 2024 (*n* = 146, 13.72%) producing most articles, and a total citation count of 32,245 over the period. The United States (*n* = 497) and United Kingdom (*n* = 131) produced the most scientific articles, while leading researchers were Mantzios, M. (*n* = 26) and Tylka, T. L. (*n* = 25). The most cited articles focused on mindfulness or meditation‐based therapies in managing psychological stress and the influence of taste on food choices.

**Conclusion:**

This study highlights a growing interest in mindful and intuitive eating, particularly with psychological and clinical applications, and emphasizes the need for stronger interdisciplinary research and international collaboration to guide future research and support the development of more culturally informed interventions.

**Abbreviations**
IE: intuitive eating
MCP: multiple countries publication
ME: mindful eating
SCP: single‐country publication
TC: total citation

## 1 Introduction

Eating behavior is used to respond to hunger to regulate internal homeostasis but problematic eating behaviors such as emotional eating or binge eating can lead to excessive food intake [1, 2]. Problematic eating behavior can stem from a number of biological, individual, and social factors including negative emotions like stress, low self‐esteem, bullying and peer pressure, body dissatisfaction and dieting, poverty and food insecurity, and the availability of low‐cost high‐calorie food and ultra‐processed food. This can lead to increased body weight and obesity along with other physical and mental health issues [3, 4]. For example, a systematic review found that ultra‐processed food consumption was associated with a range of noncommunicable diseases, including abdominal obesity, metabolic syndrome, and depression in adults [5].

Obesity is a global public health problem with the World Health Organization reporting one in eight people in the world live with obesity, approximately 890 million adults and 160 million children and adolescents [6]. At the individual level, living with obesity is a risk factor for several chronic illnesses including Type 2 diabetes, cancer, cardiovascular diseases, and neurological disorders among others [6]. These conditions can further increase the risk of morbidities and mortality [7, 8]. The global economic impact associated with overweight and obesity is substantial, with estimates suggesting costs may reach $3 trillion per year by 2030 [6].

Traditional weight management methods for tackling unhealthy eating habits such as restricting calorie intake or limiting certain types of food may be effective in the short‐term but can have negative effects on both physical and mental health in the long‐term [9, 10]. For example, the ketogenic diet requires strict control of carbohydrate intake to promote weight loss [11]. Although this method can effectively reduce weight in the short‐term, long‐term effects of low‐carbohydrate diets are limited and may lead to deficiencies in vitamins and minerals and also affect cardiovascular health [12]. Additionally, prolonged restriction of carbohydrates can affect serotonin levels in the brain, potentially leading to depression and increased anxiety [13].

Alternative strategies for addressing unhealthy eating habits have emerged such as mindful eating (ME) and intuitive eating (IE). Although there is no universally accepted definition of ME, it has been characterized by in the moment attention to eating, along with nonjudgmental awareness of physical and emotional sensations when eating [14]. The focus on paying attention to hunger and satiety signals may foster a healthier relationship with food and reduce the risk of binge eating [15]. Although ME is rooted in mindfulness and focuses on cultivating present, nonjudgmental awareness during eating episodes, IE is an adaptive eating style that emphasizes reconnecting with natural physiological signals and satiety cues and advocates for giving up any dietary restrictions or rules about healthy or unhealthy eating [16]. It may help individuals decrease excessive focus on weight, emphasizing the importance of respecting the body's physiological needs for food, rather than relying on external cues such as emotions and food availability to eat [15, 17]. Although neither ME nor IE promote intentional weight loss, both approaches are increasingly situated within weight‐related health research because they target eating behaviors such as emotional eating, binge eating, and dysregulated responses to hunger and satiety that contribute to long‐term energy balance [18, 19]. Therefore, understanding the scientific evolution of ME and IE can help explore alternative, nondiet behavioral strategies that foster healthier relationships with food and potentially support long‐term weight‐related health outcomes [20, 21].

A number of scientific reviews examining the effectiveness of mindfulness, ME, and IE on eating behaviors have been conducted over the last decade, with some showing that mindfulness‐based approaches reduced problematic eating patterns although their effects on weight loss were mixed [22, 23]. Furthermore, there is limited evidence that ME and IE based programs can influence dietary intake in adults with no history of eating disorders [24]. Given the growing volume of literature on ME and IE, a historical analysis of the contribution these approaches make to science on weight management would be helpful to highlight the major trends and knowledge gaps to support future research and practice.

### 1.1 Bibliometrics

Bibliometric analysis offers a quantitative approach to analyzing a body of literature within a particular scientific field, allowing an exploration of key concepts and research trends in a given topic [25]. It usually encompasses a review of scholarly literature, the analysis of citation and publication patterns, and the visualization of connections between different areas of research. For instance, bibliometrics has been used in the field of weight management to summarize topics like diabetes mellitus and bariatric surgery [26], global obesity research [27], and sarcopenic obesity [28], among others. This revealed important insights including notable publication trends, seminal research studies in these fields, the most prolific authors, research collaborations and disciplinary contributions, and the growth of certain research trends over time, enabling knowledge gaps to be identified and areas for further research to be recommended. However, no such analysis has been undertaken on ME and IE‐related publications. This would help uncover important scientific trends in this area that could help advance weight management practice by professionals and patients and identify areas for further research. Hence, our aim for this bibliometric study is to quantify, describe, and compare published research on ME and IE over the past 20 years.

## 2 Materials and Methods

Our research team consisted of two mindfulness practitioners and researchers (AB and TE) and three medical informatics researchers who were also registered nurses (MZ, ES, and SO). We used the BIBLIO checklist to enhance the transparency of reporting this bibliometric review [3] (File S1).

### 2.1 Search Strategy and Data Collection

Using a single, comprehensive database is a common and widely accepted practice in bibliometric studies, particularly when the aim is to analyze publication patterns, citation structures, and research networks [28, 29]. Hence, we utilized the Scopus database as it is widely recognized as a reliable and comprehensive source of academic resources that span a wide range of influential areas in relation to health, life, physical and social sciences, and the humanities [30, 31]. Furthermore, due to its broad coverage, it has been used to conduct bibliometric analysis in the field of obesity [32, 33]. We retrieved data from Scopus, covering publications from 2004 to 2024. We restricted the search to two terms “mindful* or intuit*” AND “eat*” within the title, abstract, and keyword fields to ensure relevance to mindful or intuitive eating, with the search conducted in August 2024. We set language restrictions to English and specified document types as “Article” and “Reviews,” resulting in a total of 1922 articles.

We exported bibliometric data in .CSV and .RIS formats, including citation information, bibliographical information, abstract and keywords, and cited references. We used Covidence (www.covidence.org) to screen titles and abstracts to assess the eligibility of research studies based on pre‐defined eligibility criteria (SO and MZ). We set the inclusion criteria as English‐language publications and peer‐reviewed articles and reviews that explicitly examined ME or IE concepts, and the exclusion criteria as publication types such as conference abstracts, letters, book chapters, and research studies in which ME or IE were not core constructs.

### 2.2 Data Analysis and Visualizations

We extracted data including authors, year, country, institutional affiliation, journals, titles, abstracts, author keywords, index keywords, and reference lists and performed descriptive statistics using Microsoft Excel. We used two tools to produce the bibliometric analysis: (1) Bibliometrix (https://www.bibliometrix.org/, R studio was downloaded to facilitate analysis), and (2) VOSviewer 1.6.20 (https://www.vosviewer.com/). Bibliometrix is an R package that enables visualization and network analysis, helping users explore research trends, collaborations, and impact within a scientific field [34], whereas VOSviewer is free visualization software that facilitates a range of bibliometric analyses including co‐authorship analysis, co‐occurrence analysis, citation analysis, bibliographic coupling analysis, and cocitation analysis [35].

In the analysis, we used total link strength which refers to the cumulative strength of all the connections a given entity has with others in a network, a measure to quantify the intensity or frequency of collaborations [35]. Furthermore, the “most cited articles” and “most cited references” have different meanings. The *most cited articles* refer to papers that have been cited the most by other studies, reflecting the academic influence of these studies within the given field, helping to identify key research and trends in a specific area. On the other hand, the *most cited references* refer to references frequently cited within the analyzed documents, showcasing the commonly cited research studies in the field [34]. A combined analysis of the two metrics helps to comprehensively understand the influence distribution and scientific knowledge base in a specific field. We did not require ethical approval for this study as all data were sourced from a public database for secondary analysis.

## 3 Results

After screening titles and abstracts, a total of 1064 studies were found over the 20‐year period, with the first published studies referring to meditation, mindfulness, or intuitive eating appearing in 1997 [36] and 1998 [37]. The number of studies increased most years, with 2023 (*n* = 143, 13.44%) and 2024 (*n* = 146, 13.72%) producing the highest volumes of articles (Table 1). From 1997 to 2024, the citation count increased from 75 citations in 1997 to a peak of 3676 citations in 2013, a total of 32,245 citations for the whole period. In 2022 and 2024, there was a downward trend in total citations, from 1484 to 916 and citations per publication from 4.60 to 2.84. This may reflect the length of time it can take for research studies to be cited and citation counts to accrue in a given field [38]. The average citation per publication ranged from 0.10 in 1998 to 11.40 in 2011.

**TABLE 1:** Publication metrics in ME or IE research from 1997 to 2024.

Year	Total publications (*n*, %)	Total number of citations	Average citations per publication
2004	4 (0.38)	80	0.25
2005	4 (0.38)	622	1.93
2006	7 (0.66)	1921	5.96
2007	4 (0.38)	374	1.16
2008	9 (0.85)	1739	5.39
2009	10 (0.94)	1012	3.14
2010	13 (1.22)	3303	10.24
2011	19 (1.79)	1462	4.53
2012	28 (2.63)	1427	4.43
2013	30 (2.82)	3676	11.40
2014	32 (3.01)	1930	5.99
2015	40 (3.76)	1710	5.30
2016	51 (4.79)	2504	7.77
2017	53 (4.98)	2313	7.17
2018	65 (6.11)	1777	5.51
2019	85 (7.99)	1312	4.07
2020	97 (9.12)	1745	5.41
2021	110 (10.34)	46	0.14
2022	111 (10.43)	1484	4.60
2023	143 (13.44)	345	1.07
2024	146 (13.72)	916	2.84

### 3.1 Country Analysis

The analysis of country production and citation counts highlights the dominant role of the United States across multiple metrics in the ME and IE research field. In terms of corresponding author contributions, the United States was the top ranked country, indicating a robust domestic research output (*n* = 376) and extensive international collaborations, with single‐country publication (SCP) at 342 and multiple country publication (MCP) at 34 (Figure 1). This is supported by the “Most Cited Countries” data (Figure 2), where the United States stands out with the highest number of citations (*n* = 16,361), reflecting the widespread influence and recognition of this country's research in the field. The United Kingdom ranks second and shows a substantial number of outputs (*n* = 93) and citations (*n* = 2318), emphasizing its role in international research networks. Canada ranks third in corresponding author's number of articles (*n* = 58), demonstrating its influence and active participation in ME and IE research. High‐income countries such as Australia, the Netherlands, Germany, Portugal, New Zealand, Italy, and France consistently appear in the top ranks for both article production and article citations. Meanwhile, emerging countries such as China and Turkey are making notable strides, indicating their increasing involvement in this global research community (Table 2).

**FIGURE 1:**
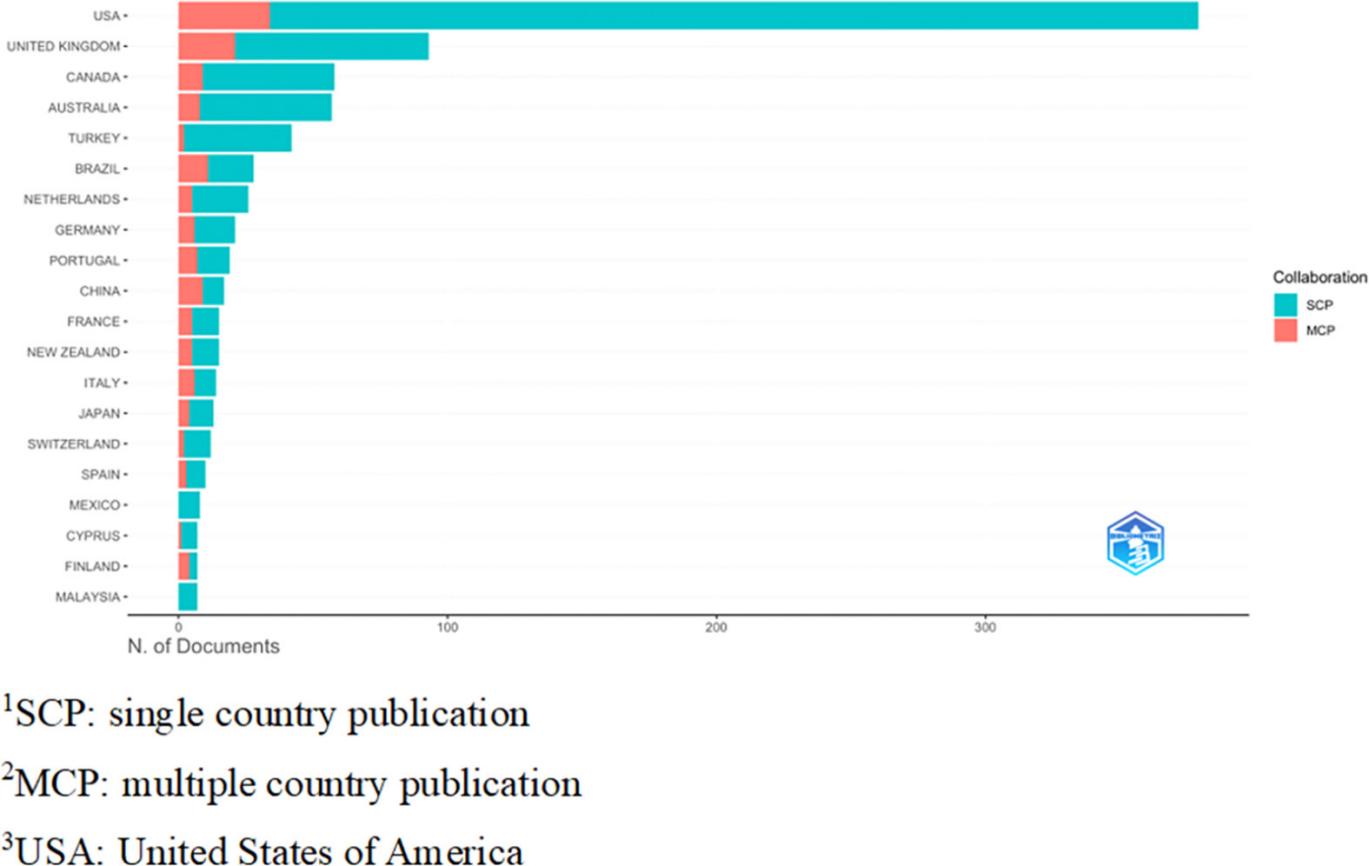
Number of publications and collaborations by corresponding author's countries.

**FIGURE 2:**
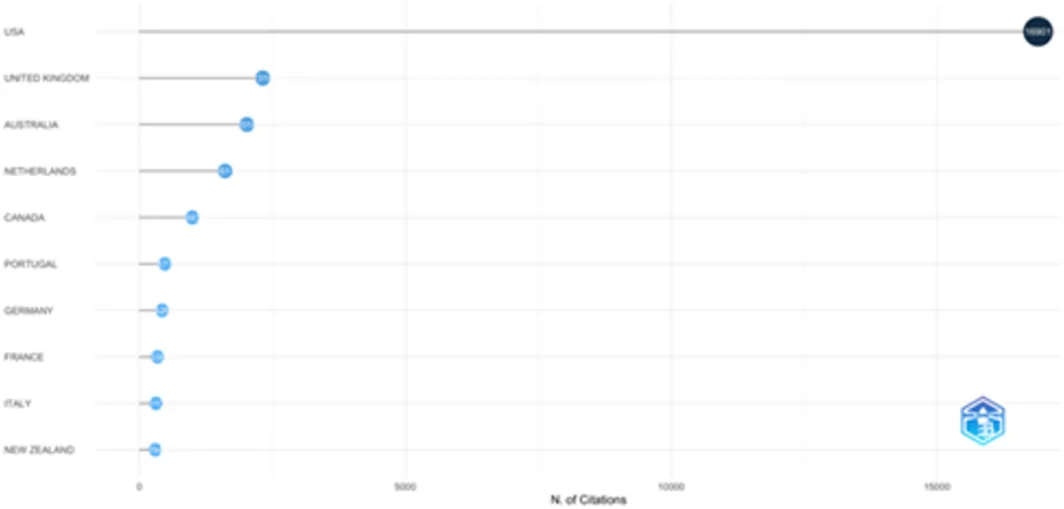
Top 10 most cited countries by corresponding author.

**TABLE 2:** Top countries by corresponding authors.

Country	Articles	Articles %	SCP	MCP	MCP %
The United States	376	36.5	342	34	9
The United Kingdom	93	9	72	21	22.6
Canada	58	5.6	49	9	15.5
Australia	57	5.5	49	8	14
Turkey	42	4.1	40	2	4.8
Brazil	28	2.7	17	11	39.3
The Netherlands	26	2.5	21	5	19.2
Germany	21	2	15	6	28.6
Portugal	19	1.8	12	7	36.8
China	17	1.7	8	9	52.9

Abbreviations: MCP, multiple country publication; SCP, single‐country publication; UK, United Kingdom; USA, United States.

Figure 3 further illustrates publication trends in ME and IE, with the United States showing a steep increase in ME and IE research paper production over the past two decades. This growth trajectory underscores its expanding research capacity and leadership in scientific contributions in these fields. Other countries like Canada, Brazil, Australia, and the United Kingdom display steady growth in research output, highlighting their sustained scholarship and collaboration.

**FIGURE 3:**
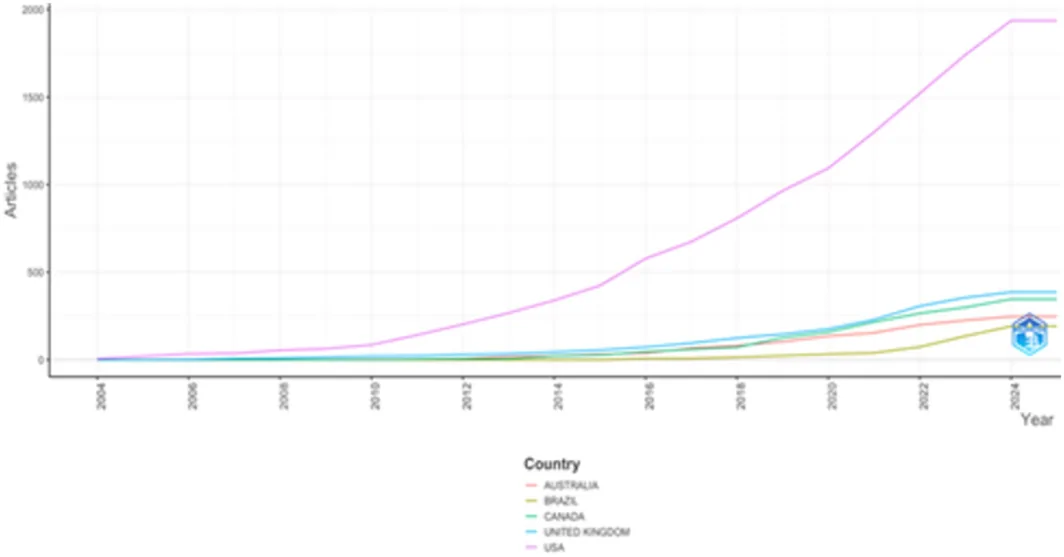
Top 5 countries article production over time.

### 3.2 Co‐Authorship Analysis

Co‐authorship analysis is a method of network analysis used to explore the collaboration patterns between authors of academic publications [39]. Co‐authorship using country as the unit of analysis examines the collaborative relationships between different countries based on shared authorship in academic publications. This analysis helps to identify international collaborations in ME and IE research and shows how these networks are distributed globally (Figure 4).

**FIGURE 4:**
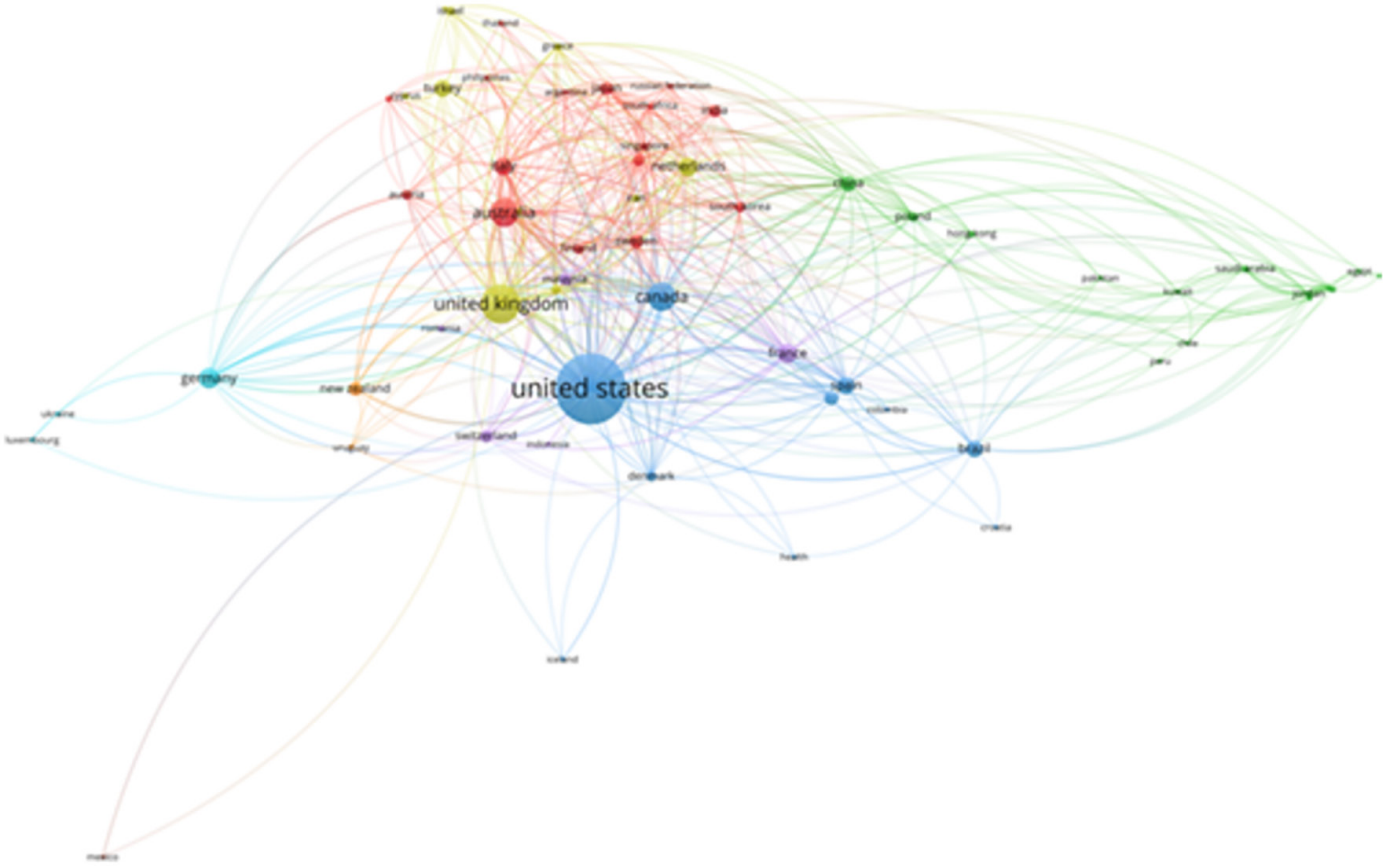
Countries with co‐authorship links (five or more publications).

Table 3 shows the top 10 countries, which play significant roles in international academic collaborations, including data on publication, citations, and total link strength. The United States has the highest total link strength of 141 (with 497 publications and 19,780 citations), indicating its central position in the global ME and IE research network and its substantial academic influence. The United Kingdom follows, with 131 publications and 4558 citations, and a total link strength of 91. High‐income countries, such as Canada, Australia, France, Italy, Spain, Germany, and Japan are also prominent in the co‐authorship network, highlighting their important roles in global research partnerships. China, as the only low‐ and middle‐ income country, also demonstrates active participation in international research in this field.

**TABLE 3:** Co‐authorship analysis (Top 10 countries with highest total link strength).

Rank	Country	Publications	Citations	Total link strength
1	The United States	497	19,780	141
2	The United Kingdom	131	4558	91
3	Canada	88	1447	68
4	Australia	78	2518	53
5	China	23	189	43
6	France	26	578	40
7	Italy	24	483	40
8	Spain	19	340	36
9	Germany	40	871	31
10	Japan	17	129	24

### 3.3 Author Analysis

In total, 3467 authors have contributed to ME or IE‐related research. The top 10 most productive authors are shown in Table 4, with Mantzios as the leading author having 26 publications and 494 citations, boasting a substantial total link strength of 51. Tylka follows with 25 publications and 2340 citations, with total link strength of 18, and Egan is the third most prolific author with 21 publications, 252 citations, and a total link strength of 48. Figure 5 highlights trends in research productivity among the top 10 most productive authors over time which shows that authors such as Kristeller, Hecht, and Tylka have maintained sustained publication outputs in ME and IE over the years, with a noticeable increase in publication activity in ME and IE‐related research around 2017–2018 for several authors.

**TABLE 4:** Top 10 most productive authors in ME and IE research.

Rank	Author name	Publications	Citations	Total link strength
1	Mantzios, M.	26	494	51
2	Tylka, T. L.	25	2340	18
3	Egan, H.	21	252	48
4	Neumark‐Sztainer, D.	16	424	26
5	Keyte, R.	13	175	34
6	Linardon, J.	14	587	19
7	Provencher, V.	13	219	20
8	Hussain, M.	13	156	38
9	Kristeller, J.	12	893	56
10	Hecht, F. M.	10	755	58

**FIGURE 5:**
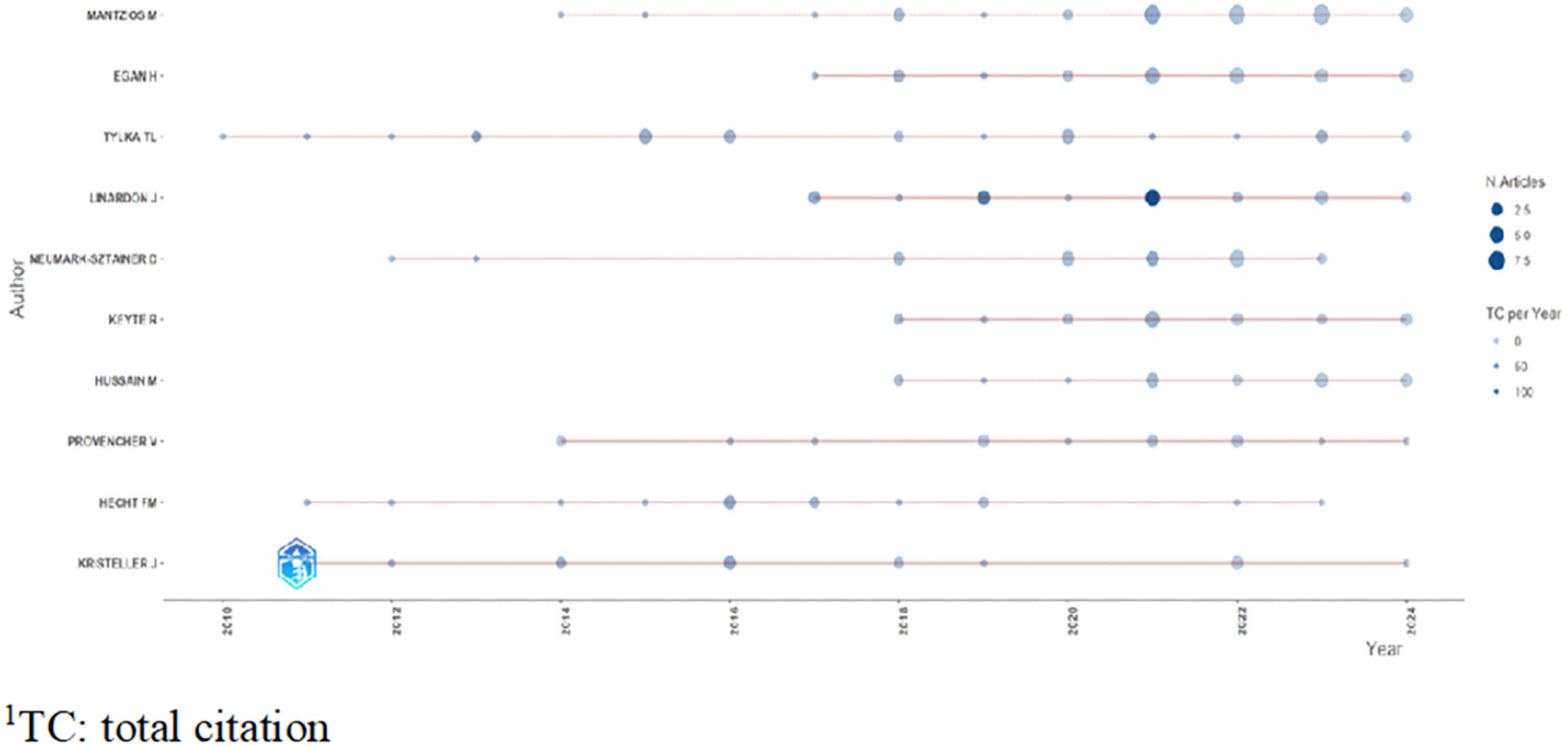
Top 10 productive authors' productivity over time (TC—total citations).

### 3.4 Citation Analysis

The top 10 most cited articles on ME or IE research show a wide range of citation counts from 336 to 2590. The highest citation count is 2590, attributed to the paper titled “The effect of mindfulness‐based therapy on anxiety and depression: A meta‐analytic review” published in the Journal of Consulting and Clinical Psychology in 2010 (Table 5). Of the 1064 retrieved articles, a total of 43,196 references were cited. Table 6 presents the top 10 most cited references in the dataset, showcasing the most influential research on ME and IE.

**TABLE 5:** Top 10 cited articles in ME or IE research.

Rank	Article title	Year	Source	Citations
1	The effect of mindfulness‐based therapy on anxiety and depression: A meta‐analytic review	2010	*Journal of Consulting and Clinical Psychology*	2590
2	Meditation programs for psychological stress and well‐being	2014	*JAMA Internal Medicine*	1460
3	The unhealthy = tasty intuition and its effects on taste inferences, enjoyment, and choice of food products	2006	*Journal of Marketing*	908
4	Diversity in the determinants of food choice: A psychological perspective	2009	*Food Quality and Preference*	662
5	Self‐enhancement: food for thought	2008	*Perspectives on Psychological Science*	563
6	The intuitive eating scale–2: Item refinement and psychometric evaluation with college women and men	2013	*Journal of Counseling Psychology*	462
7	Development and validation of a self‐report measure of mentalizing: The reflective functioning questionnaire	2016	*PLOS One*	441
8	Mindfulness‐based eating awareness training for treating binge eating disorder: the conceptual foundation	2014	Eating Disorder and Mindfulness	376
9	Development and psychometric evaluation of a measure of intuitive eating	2006	*Journal of Counseling Psychology*	366
10	Mindfulness‐based interventions for obesity‐related eating behaviors: a literature review	2014	Obesity Reviews	336

**TABLE 6:** Top 10 cited references in ME or IE research.

Rank	Article title	Year	Source	Citations
1	A systematic review of the psychosocial correlates of intuitive eating among adult women	2016	Appetite	76
2	Development and psychometric evaluation of a measure of intuitive eating	2006	*Journal of Counseling Psychology*	58
3	The Body Appreciation Scale‐2: item refinement and psychometric evaluation	2015	Body Image	51
4	Does mindfulness matter? Everyday mindfulness, mindful eating and self‐reported serving size of energy dense foods among a sample of South Australian adults	2013	Appetite	49
5	Statistical Power Analysis for the Behavioral Sciences	1988	Taylor & Francis	48
6	Is intuitive eating the same as flexible dietary control? Their links to each other and well‐being could provide an answer	2015	Appetite	46
7	Using self‐report assessment methods to explore facets of mindfulness	2006	Assessment	44
8	A structured literature review on the role of mindfulness, mindful eating and intuitive eating in changing eating behaviors: effectiveness and associated potential mechanisms	2017	Nutrition research reviews	39
9	Diagnostic and statistical manual of mental disorders 5: A quick glance	2013	*Indian Journal of Psychiatry*	38
10	Development and validation of the mindful eating questionnaire	2009	*Journal of the American Dietetic Association*	38

### 3.5 Keywords Analysis

#### 3.5.1 Cluster Analysis

We used VOSviewer to conduct the co‐occurrence analysis using author keyword as the unit of analysis as they typically reflect the research theme of the article more directly, whereas index keywords are often provided by databases and tend to focus on broader subject classifications, which may not accurately capture the specific research topics [40]. There were 3647 author keywords across all publications and the analysis shows the most commonly occurring keywords (minimum occurrence of 5) in the fields of ME and IE research (Figure 6). These are grouped into nine clusters in the figure, with each dot representing an author keyword and the size of dots reflecting the frequency of occurrences in the dataset. Among the top three colored clusters, the red cluster has 45 keywords, 119 occurrences, and 98 links, with a total link strength of 298. Within this cluster, the top keywords associated with eating disorders are mental health, anorexia nervosa, binge eating, and therapies such as acceptance and commitment therapy and psychotherapy. The green cluster has 35 keywords, 51 occurrences, and 74 links, with total link strength of 189. Within this cluster, the top keywords associated with weight loss are physical activity, nutrition, and healthy eating. The blue cluster has 35 keywords, 296 occurrences, and 158 links, with total link strength of 705. Within this cluster, the top keywords associated with intuitive eating are body image, body dissatisfaction, body appreciation, and positive body image. The most frequent author keyword co‐occurrences include “mindfulness,” “intuitive eating,” and “obesity” (Table 7).

**FIGURE 6:**
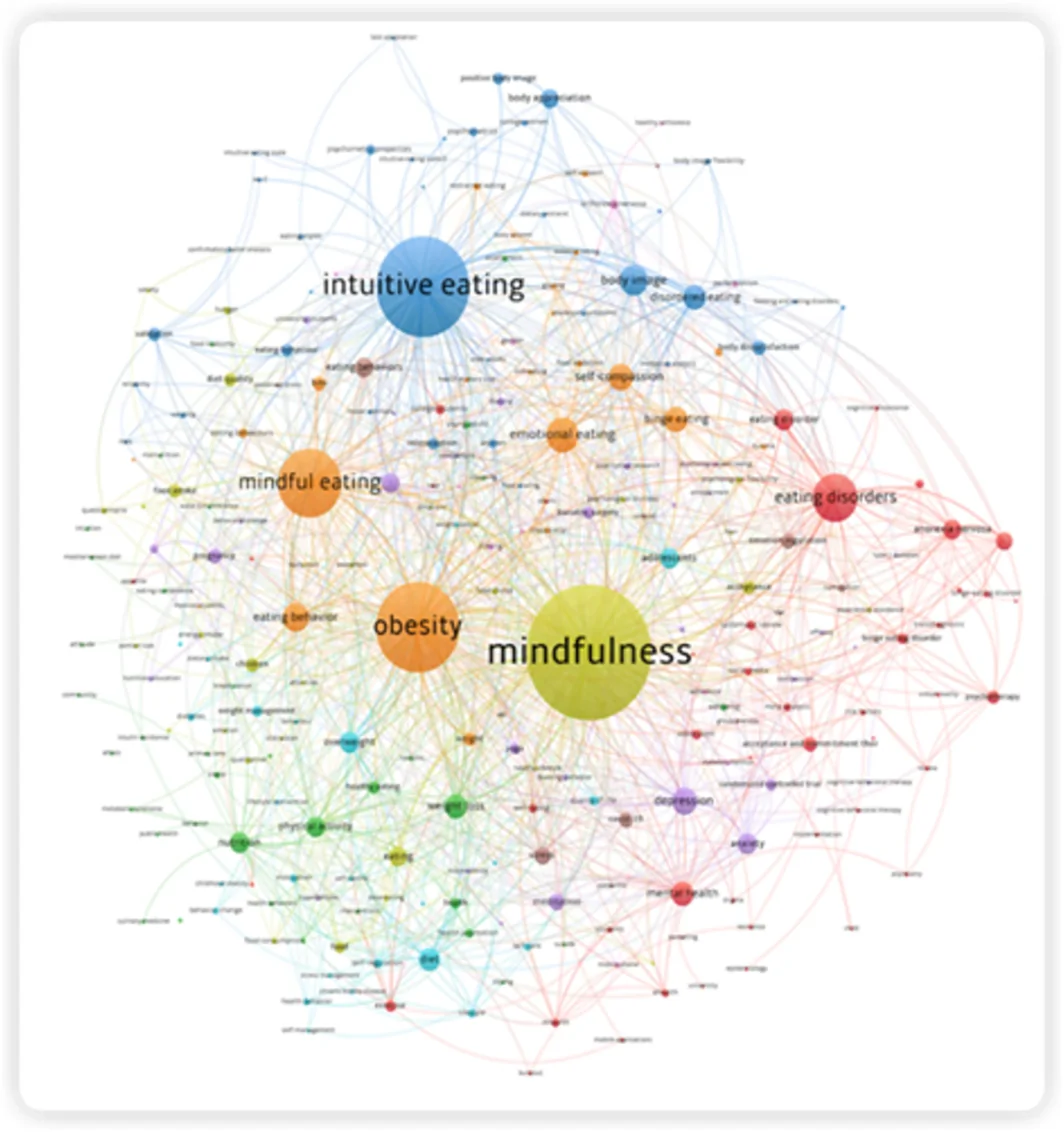
Network visualization of author keywords (minimum occurrence of 5).

**TABLE 7:** Top 10 author keyword co‐occurrence.

Rank	Author keyword	Occurrences	Total link strength
1	Mindfulness	429	1185
2	Intuitive eating	296	714
3	Obesity	258	816
4	Mindful eating	184	475
5	Eating disorders	119	313
6	Emotional eating	80	251
7	Body image	68	209
8	Eating behavior	62	182
9	Depression	59	197
10	Self‐compassion	57	157

#### 3.5.2 Trend Analysis

We also used Bibliometrix to analyze research trends related to ME and IE in the past decade. As shown in Figure 7, the evolution of topics such as “mindfulness‐based cognitive therapy” and *drug therapy* has become popular in recent years. Core issues like *homeostasis*, *appetite*, and *psychological aspects* have sustained attention throughout the entire timeframe, indicating their long‐term significance. Emerging areas of focus include *intuitive eating* and *self‐compassion*, which have gained more prominence in recent years. The research also reflects diversity in subjects, covering various age groups including *young adult* and *middle‐aged* adults and genders.

**FIGURE 7:**
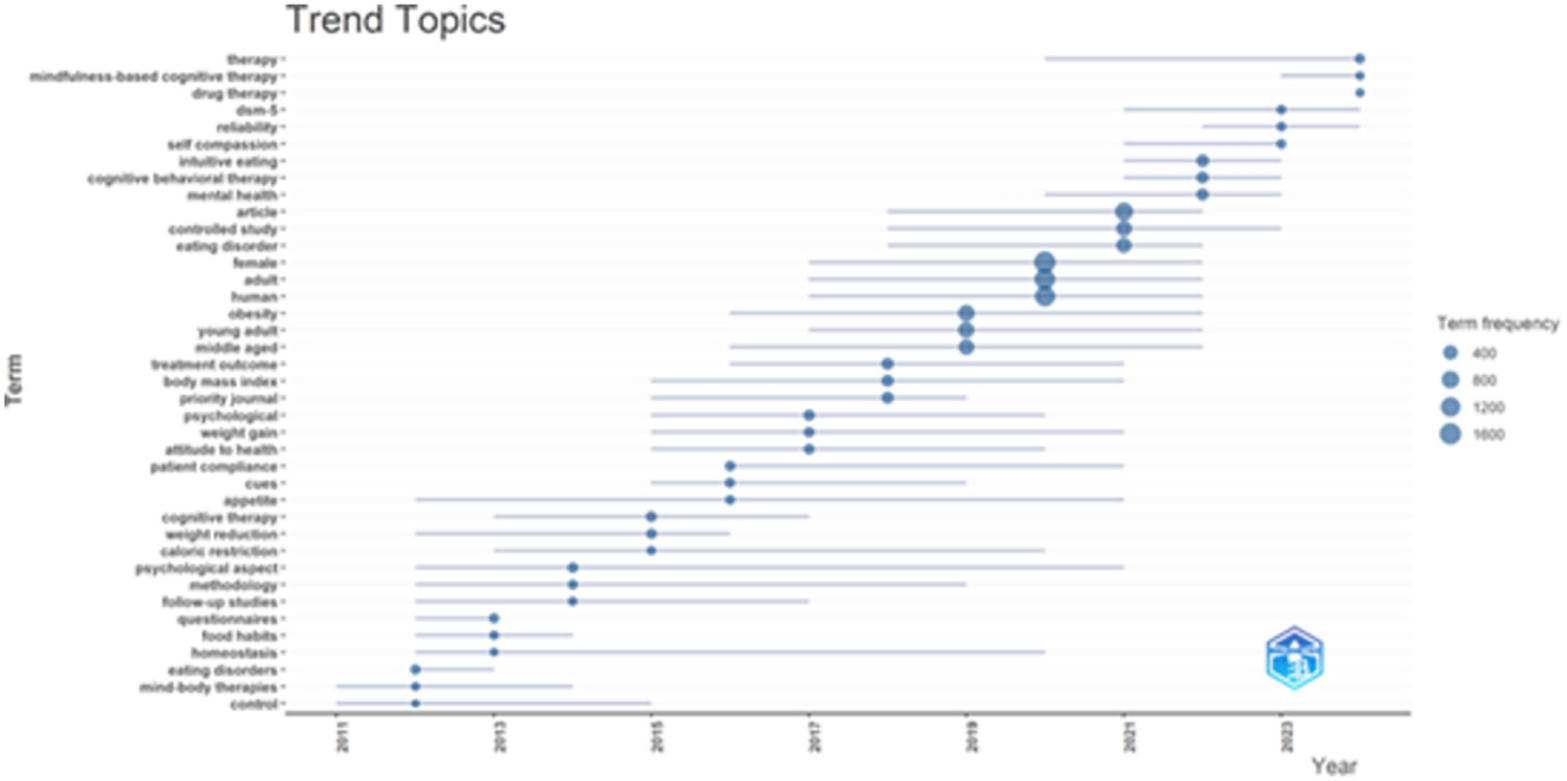
Trend topics in ME and IE (generated by Bibliometrix).

## 4 Discussion

### 4.1 Principal Findings

This study provides the most updated analysis on ME and IE research over the last two decades. Interest in ME and IE has risen steadily since 2004, with publication and citation activity reaching a clear peak between 2010 and 2020. However, citation patterns in the most recent years should be interpreted with caution as with most bibliometric analyses, recent publications simply have not had enough time to accumulate citations [41]. Therefore, citation indicators for these years are best understood as reflecting publication volume rather than impact. Interestingly, commonly used keywords related to eating disorders, weight loss, and obesity appear increasingly linked to ME and IE, despite these approaches originally aiming to foster healthier relationships with food rather than promoting weight control [15]. This shift suggests that ME and IE are being progressively integrated into clinical and behavioral health contexts to address complex eating‐related concerns and weight control problems.

Productivity and co‐authorship analysis results on countries, authors, and institutions highlight the United States as the dominant force in global research on ME and IE, leading in both publication and citation counts, as well as extensive international collaborations. The United Kingdom and Australia also play significant roles, reflecting strong participation in academic networks. Notably, European countries consistently rank high in research output, although citations with emerging nations, particularly China and Turkey, are increasing their influence in global research on this topic. This distribution also reveals that low‐ and middle‐income countries remain underrepresented within the fields of ME and IE, reflecting broader disparities in research capacity, funding, and cross‐national collaboration [42]. The analysis of co‐authorship reveals robust collaboration patterns, with the United States showing the highest total link strength, followed by the United Kingdom and Canada. Although mindfulness developed from Buddhist traditions in Eastern philosophy [43], most research in this review originated in Western countries, which have yet to adopt ME and IE practices in their eating cultures. Hence, more multicultural collaboration could enhance research by not only improving the development of ME and IE interventions but also outcome measures.

Trend analysis shows that psychological aspects, assessment tools, research methodology, and methods have gained increasing attention, reflecting the dynamic progression on ME and IE research. The cluster analysis reveals nine clusters in ME and IE research, with emerging areas like *intuitive eating*, *mindful eating*, and *mindfulness* gaining prominence over time. The most influential articles in the fields of ME and IE were also identified, which can help researchers identify research hotspots and relevant concepts and theories. These most influential articles point toward a shared theoretical direction, with many drawing on ideas around self‐regulation and psychological processes that shape eating behaviors. This may explain why certain clusters, such as “mindfulness,” “mindful eating,” and “intuitive eating,” have become increasingly prominent.

Other theories such as self‐determination theory, a humanistic psychological framework that centres on intrinsic motivation as a driver for satisfying three core psychological needs (i.e., autonomy, competence, and relatedness) [44], are also being used to understand eating behaviors and weight change with emerging evidence they could support body satisfaction and healthy eating habits [45]. Therefore, these types of theories may be useful when developing and evaluating mindful and intuitive eating interventions given the links between mindfulness and motivation [46]. However, critics of behavior change theory point to the importance of the social determinants of health which span numerous socio‐economic, cultural, and geopolitical factors that can impact healthy eating and weight management in individuals and communities across the globe [47, 48]. Hence, ME and IE could be considered as one aspect of a multifaceted approach to supporting individuals with excessive body weight and problematic eating behaviors and should be balanced with public health initiatives promoting healthy eating, physical activity, and positive mental health [49].

### 4.2 Future Implications

This bibliometric analysis of ME and IE research over the past two decades provides valuable insights for future research directions. First, the dominance of countries like the United States, United Kingdom, and Australia highlights the importance of fostering international collaborations, especially with emerging nations which are increasingly contributing to global research. Expanding partnerships between developed and developing countries could diversify research perspectives and lead to advances in scientific research in ME and IE [50]. Future research efforts should prioritize building global networks to foster knowledge exchange between different nations and institutions, which could potentially elevate research impact in these fields on a worldwide scale.

Second, keyword analysis reveals important emerging areas such as *mindfulness*, *mindful eating*, and *intuitive eating*, along with growing attention to psychological aspects of IE and ME. Future studies could explore and expand these topics further. The increasing diversity of research subjects, methodologies, and methods in this research area also suggests that it is important to strengthen interdisciplinary research and integrate insights from diverse disciplines. By addressing these gaps and expanding international and interdisciplinary collaborations, researchers could strengthen the overall impact of ME and IE research in academic and real‐world settings.

### 4.3 Strengths and Limitations

A key strength of this study lies in the application of comprehensive bibliometric software including Bibliometrix and VOSviewer, which effectively captured the overall landscape of ME and IE research, illustrating key trends in these fields. However, there are some limitations to consider. First, a key limitation of this review is the restriction to English‐language publications, which may introduce geographic and cultural biases [51]. Consequently, important contributions published in other languages, particularly those grounded in non‐Western eating traditions or local health paradigms, may not be reflected in the bibliometric patterns reported here. Hence, the trends identified in these analyses should therefore be interpreted with consideration of this linguistic bias, which may constrain the extent to which the findings represent the full global landscape of ME and IE research. Second, whereas Scopus was chosen as the primary database, using other databases like Web of Science or PubMed could potentially produce different results due to differences in indexing and coverage. Third, the information extracted by bibliometric analysis software cannot guarantee 100% accuracy, such as authors' names. This may be because authors' names can appear in different forms across various articles, leading to dispersed data. Although the authors of this review manually cleaned and standardized the data before undertaking the analysis, inaccuracies may still occur. Lastly, although this bibliometric analysis provides useful insights into publication trends and metrics, it does not assess the quality of the included studies.

## 5 Conclusion

In this review, valuable insights into the research landscape of ME and IE are gained through a comprehensive analysis of countries, institutions, authors, citations, journals, and keywords. By examining collaboration networks between countries, institutions, and authors, as well as identifying emerging trends through keyword analysis, the study highlights the current state of research in these fields. The results offer a clear understanding of the research dynamics in the past two decades, enabling researchers to identify key trends and potential collaborations for future studies on ME and IE.

## Author Contributions

Siobhan O'Connor and Mengying Zhang designed and conducted the study, collated and analyzed the data, and Mengying Zhang drafted the manuscript. Angeliki Bogossian, Emma Stanmore, and Trudi Edginton assisted in the visualization of the bibliometric analysis, provided research expertise, and edited the manuscript. As this work's guarantor, Siobhan O'Connor has full access to all study dataset and is responsible for the data's integrity and accuracy.

## Conflicts of Interest

The authors declare no conflicts of interest.

## Supporting information


**Data S1:** The BIBLIO checklist for reporting the bibliometric reviews of the biomedical literature.

## Data Availability

The data that support the findings of this study are available from the corresponding author upon reasonable request.

## References

[1] L. Cifuentes and A. Acosta , “Homeostatic Regulation of Food Intake,” Clinics and Research in Hepatology and Gastroenterology 46, no. 2 (2022): 101794. 10.1016/j.clinre.2021.101794 PMC9721532 34481092

[2] J. Reichenberger , R. Schnepper , A. K. Arend , et al., “Emotional Eating Across Different Eating Disorders and the Role of Body Mass, Restriction, and Binge Eating,” International Journal of Eating Disorders 54, no. 5 (2021): 773–784. 10.1002/eat.23477 PMC8252459 33656204

[3] A. Montazeri , S. Mohammadi , P. M. Hesari , M. Ghaemi , H. Riazi , and Z. Sheikhi‐Mobarakeh , “Preliminary Guideline for Reporting Bibliometric Reviews of the Biomedical Literature (BIBLIO): A Minimum Requirements,” Systematic Reviews 12, no. 1 (2023): 239 –210. 10.1186/s13643-023-02410-2 PMC10722750 38102710

[4] L. Kudlek , P. Eustachio Colombo , A. Ahern , et al., “The Impact of Behavioral Weight Management Interventions on Eating Behavior Traits in Children With Overweight or Obesity: Systematic Review and Meta‐Analysis,” Obesity Reviews 26, no. 1 (2025): e13839. 10.1111/obr.13839 PMC11611439 39299797

[5] G. Pagliai , M. Dinu , M. P. Madarena , M. Bonaccio , L. Iacoviello , and F. Sofi , “Consumption of Ultra‐Processed Foods and Health Status: A Systematic Review and Meta‐Analysis,” British Journal of Nutrition 125, no. 3 (2021): 308–318. 10.1017/S0007114520002688 PMC7844609 32792031

[6] WHO , Obesity and Overweight (WHO, 2024), https://www.who.int/news‐room/fact‐sheets/detail/obesity‐and‐overweight.

[7] Y. C. Chooi , C. Ding , and F. Magkos , “The Epidemiology of Obesity,” Metabolism 92 (2019): 6–10, 10.1016/j.metabol.2018.09.005. 30253139

[8] M. Safaei , E. A. Sundararajan , M. Driss , W. Boulila , and A. Shapi'i , “A Systematic Literature Review on Obesity: Understanding the Causes & Consequences of Obesity and Reviewing Various Machine Learning Approaches Used to Predict Obesity,” Computers in Biology and Medicine 136 (2021): 104754, 10.1016/j.compbiomed.2021.104754. 34426171

[9] G. D. Brinkworth , “Long‐Term Effects of a Very Low‐Carbohydrate Diet and a Low‐Fat Diet on Mood and Cognitive Function,” Archives of Internal Medicine 169, no. 20 (2009): 1873–1880. 10.1001/archinternmed.2009.329 19901139

[10] E. Daneshzad , S. A. Keshavarz , M. Qorbani , B. Larijani , and L. Azadbakht , “Association Between a Low‐Carbohydrate Diet and Sleep Status, Depression, Anxiety, and Stress Score,” Journal of the Science of Food and Agriculture 100, no. 7 (2020): 2946–2952. 10.1002/jsfa.10322 32031258

[11] B. O'Neill and P. Raggi , “The Ketogenic Diet: Pros and Cons,” Atherosclerosis 292 (2020): 119–126, 10.1016/j.atherosclerosis.2019.11.021. 31805451

[12] G. A. Silverii , C. Cosentino , F. Santagiuliana , et al., “Effectiveness of Low‐Carbohydrate Diets for Long‐Term Weight Loss in Obese Individuals: A Meta‐Analysis of Randomized Controlled Trials,” Diabetes, Obesity and Metabolism 24, no. 8 (2022): 1458–1468. 10.1111/dom.14709 PMC9546386 35373905

[13] M. Watanabe , D. Tuccinardi , I. Ernesti , et al., “Scientific Evidence Underlying Contraindications to the Ketogenic Diet: An Update,” Obesity Reviews 21, no. 10 (2020): e13053. 10.1111/obr.13053 PMC7539910 32648647

[14] M. Mantzios , “(Re)defining Mindful Eating Into Mindful Eating Behaviour to Advance Scientific Enquiry,” Nutrition and Health 27, no. 4 (2021): 367–371. 10.1177/0260106020984091 33356846

[15] J. M. Warren , N. Smith , and M. Ashwell , “A Structured Literature Review on the Role of Mindfulness, Mindful Eating and Intuitive Eating in Changing Eating Behaviours: Effectiveness and Associated Potential Mechanisms,” Nutrition Research Reviews 30, no. 2 (2017): 272–283. 10.1017/S0954422417000154 28718396

[16] T. L. Tylka and J. A. Wilcox , “Are Intuitive Eating and Eating Disorder Symptomatology Opposite Poles of the Same Construct?,” Journal of Counseling Psychology 53, no. 4 (2006): 474–485.

[17] J. Linardon , T. L. Tylka , and M. Fuller‐Tyszkiewicz , “Intuitive Eating and Its Psychological Correlates: A Meta‐Analysis,” International Journal of Eating Disorders 54, no. 7 (2021): 1073–1098. 10.1002/eat.23509 33786858

[18] H. Morillo‐Sarto , Y. López‐Del‐Hoyo , A. Pérez‐Aranda , et al., “‘Mindful Eating’ for Reducing Emotional Eating in Patients With Overweight or Obesity in Primary Care Settings: A Randomized Controlled Trial,” European Eating Disorders Review 31, no. 2 (2023): 303–319. 10.1002/erv.2958 PMC10100015 36397211

[19] T. P. Minari , G. M. . Araújo‐Filho , L. H. B. Tácito , et al., “Effects of Mindful Eating in Patients With Obesity and Binge Eating Disorder,” Nutrients 16, no. 6 (2024): 884. 10.3390/nu16060884 PMC10975968 38542795

[20] T. S. Kao , J. Ling , M. Alanazi , A. Atwa , and S. Liu , “Effects of Mindfulness‐Based Interventions on Obesogenic Eating Behaviors: A Systematic Review and Meta‐Analysis,” Obesity Reviews 26, no. 3 (2025): e13860. 10.1111/obr.13860 PMC11791380 39489689

[21] E. R. Lawlor , N. Islam , S. Bates , et al., “Third‐Wave Cognitive Behaviour Therapies for Weight Management: A Systematic Review and Network Meta‐Analysis,” Obesity Reviews 21, no. 7 (2020): e13013. 10.1111/obr.13013 PMC7379202 32181957

[22] K. Carrière , B. Khoury , M. M. Günak , and B. Knäuper , “Mindfulness‐Based Interventions for Weight Loss: A Systematic Review and Meta‐Analysis,” Obesity Reviews 19, no. 2 (2018): 164–177. 10.1111/obr.12623 29076610

[23] D. Mercado , L. Robinson , G. Gordon , J. Werthmann , I. C. Campbell , and U. Schmidt , “The Outcomes of Mindfulness‐Based Interventions for Obesity and Binge Eating Disorder: A Meta‐Analysis of Randomised Controlled Trials,” Appetite 166 (November 2021): 105464, 10.1016/j.appet.2021.105464. 34146647

[24] H. S. Grider , S. M. Douglas , and H. A. Raynor , “The Influence of Mindful Eating and/or Intuitive Eating Approaches on Dietary Intake: A Systematic Review,” Journal of the Academy of Nutrition and Dietetics 121, no. 4 (2021): 709–727.e1. 10.1016/j.jand.2020.10.019 33279464

[25] N. Donthu , S. Kumar , D. Mukherjee , N. Pandey , and W. M. Lim , “How to Conduct a Bibliometric Analysis: An Overview and Guidelines,” Journal of Business Research 133 (2021): 285–296, 10.1016/j.jbusres.2021.04.070.

[26] K. Yuan , X. Zhang , B. Wu , R. Zeng , R. Hu , and C. Wang , “Research Trends Between Diabetes Mellitus and Bariatric Surgery Researches: Bibliometric Analysis and Visualization From 1998 to 2023,” Obesity Reviews 25, no. 6 (2024): e13730. 10.1111/obr.13730 38424660

[27] A. Khan , N. Choudhury , S. Uddin , L. Hossain , and L. A. Baur , “Longitudinal Trends in Global Obesity Research and Collaboration: A Review Using Bibliometric Metadata,” Obesity Reviews 17, no. 4 (2016): 377–385. 10.1111/obr.12372 26864566

[28] F. Petermann‐Rocha , F. Diaz‐Toro , D. Valera‐Gran , and E. M. Navarrete‐Muñoz , “Bibliometric Analysis of Research on Sarcopenic Obesity: A Review of Scientific Literature,” Obesity Reviews 25, no. 9 (2024): e13784. 10.1111/obr.13784 38807369

[29] J. Vioque , J. M. Ramos , E. M. Navarrete‐Muñoz , and M. García‐de‐la‐Hera , “A Bibliometric Study of Scientific Literature on Obesity Research in PubMed (1988–2007),” Obesity Reviews 11, no. 8 (2010): 603–611. 10.1111/j.1467-789X.2009.00647.x 19754632

[30] J. F. Burnham , “Scopus Database: A Review,” Biomedical Digital Libraries 3 (2006): 1–8, 10.1186/1742-5581-3-1. PMC1420322 16522216

[31] F. G. Montoya , A. Alcayde , R. Baños , and F. Manzano‐Agugliaro , “A Fast Method for Identifying Worldwide Scientific Collaborations Using the Scopus Database,” Telematics and Informatics 35, no. 1 (2018): 168–185.

[32] A. Aletaha , A. Soltani , and F. Dokhani , “Evaluating Obesity Publications: From Bibliometrics to Altmetrics,” Journal of Diabetes and Metabolic Disorders 20, no. 1 (2021): 391–405. 10.1007/s40200-021-00758-7 PMC8212252 34178847

[33] E. L. Corrêa , L. F. P. Cotian , J. W. Lourenço , et al., “Overview of the Last 71 Years of Metabolic and Bariatric Surgery: Content Analysis and Meta‐Analysis to Investigate the Topic and Scientific Evolution,” Obesity Surgery 34, no. 5 (2024): 1885–1908. 10.1007/s11695-024-07165-w 38485892

[34] M. Aria and C. Cuccurullo , “bibliometrix: An R‐Tool for Comprehensive Science Mapping Analysis,” Journal of Informetrics 11, no. 4 (2017): 959–975.

[35] N. J. Van Eck and L. Waltman , VOSviewer Manual. Manual for VOSviewer Version (Universiteit Leiden, 2011) 1(0).

[36] B. Roth and T. Creaser , “Mindfulness Meditation‐Based Stress Reduction: Experience With a Bilingual Inner‐City Program,” Nurse Practitioner 22, no. 3 (1997): 150–152,154,157passim. 9078521

[37] J. Gast and S. R. Hawks , “Weight Loss Education: The Challenge of a New Paradigm,” Health Education & Behavior 25, no. 4 (1998): 464–473. 10.1177/109019819802500405 9690104

[38] L. Bornmann and H.‐D. Daniel , “What Do Citation Counts Measure? A Review of Studies on Citing Behavior,” Journal of Documentation 64, no. 1 (2008): 45–80.

[39] D. De Stefano , G. Giordano , and M. P. Vitale , “Issues in the Analysis of Co‐Authorship Networks,” Quality & Quantity 45 (2011): 1091–1107, 10.1007/s11135-011-9493-2.

[40] University of Southern California , Searching Solutions: Keywords vs Indexed (University of Southern California, 2025 [cited 2025 January 14th], https://libguides.usc.edu/c.php?g=631331&p=4459632.

[41] Z. S. Morris , S. Wooding , and J. Grant , “The Answer Is 17 Years, What Is the Question: Understanding Time Lags in Translational Research,” Journal of the Royal Society of Medicine 104, no. 12 (2011): 510–520. 10.1258/jrsm.2011.110180 PMC3241518 22179294

[42] M. Zhang , L. Doi , J. Awua , H. Asare , and R. Stenhouse , “Challenges and Possible Solutions for Accessing Scholarly Literature Among Medical and Nursing Professionals and Students in Low‐and‐Middle Income Countries: A Systematic Review,” Nurse Education Today 123 (2023): 105737. 10.1016/j.nedt.2023.105737 36753870

[43] R. E. Purser and J. Milillo , “Mindfulness Revisited: A Buddhist‐Based Conceptualization,” Journal of Management Inquiry 24, no. 1 (2015): 3–24.

[44] J. Q. X. Ng , J. Y. X. Chua , M. Choolani , et al., “AI or Nay? Evaluating the Potential Use of ChatGPT (Open AI) and Perplexity AI in Undergraduate Nursing Research: An Exploratory Case Study,” Nurse Education in Practice 87 (2025): 104488. 10.1016/j.nepr.2025.104488 40730086

[45] R. A. LaCaille , S. A. Hooker , and L. J. LaCaille , “Using Self‐Determination Theory to Understand Eating Behaviors and Weight Change in Emerging Adults,” Eating Behaviors 39 (2020): 101433. 10.1016/j.eatbeh.2020.101433 32961470

[46] J. N. Donald , E. L. Bradshaw , R. M. Ryan , et al., “Mindfulness and Its Association With Varied Types of Motivation: A Systematic Review and Meta‐Analysis Using Self‐Determination Theory,” Personality & social psychology bulletin 46, no. 7 (2020): 1121–1138. 10.1177/0146167219896136 31884892

[47] M. Simone , J. Slaughter‐Acey , V. M. Hazzard , M. Eisenberg , and D. Neumark‐Sztainer , “Exploring the Intersection of Multiple Social Determinants of Health and Disordered Eating Behaviors in a Population‐Based Sample in the United States,” International Journal of Eating Disorders 55, no. 11 (2022): 1589–1602. 10.1002/eat.23795 PMC9633979 36324296

[48] C. Magny‐Normilus , J. A. Sanders , P. Underwood , and R. D. LaPlante , “Associations Between Weight Discrimination and Health Outcomes by Racial and Ethnic Groups: A Scoping Review,” Obesity Reviews 27 (2025): e70016. 10.1111/obr.70016 40983524

[49] J. Chan , P. Conroy , P. Phongsavan , D. Raubenheimer , and M. Allman‐Farinelli , “From Preschool to Policy: A Scoping Review of Recommended Interventions for a Systems Approach to Improve Dietary Intake in Early Childhood,” Obesity Reviews 26, no. 6 (2025): e13897. 10.1111/obr.13897 PMC12069171 39822041

[50] D. D. Reidpath and P. Allotey , “The Problem of ‘Trickle‐Down Science’ From the Global North to the Global South,” BMJ Global Health 4, no. 4 (2019): e001719. 10.1136/bmjgh-2019-001719 PMC6666820 31406597

[51] C. Stern and J. Kleijnen , “Language Bias in Systematic Reviews: You Only Get Out What You Put in,” JBI Evidence Synthesis 18, no. 9 (2020): 1818–1819. 10.11124/JBIES-20-00361 32925418

